# Copalyl Diphosphate Synthase Mutation Improved Salt Tolerance in Maize (*Zea mays*. L) via Enhancing Vacuolar Na^+^ Sequestration and Maintaining ROS Homeostasis

**DOI:** 10.3389/fpls.2020.00457

**Published:** 2020-05-13

**Authors:** Yushi Zhang, Yubin Wang, Jiapeng Xing, Jiachi Wan, Xilei Wang, Juan Zhang, Xiaodong Wang, Zhaohu Li, Mingcai Zhang

**Affiliations:** ^1^College of Agronomy and Biotechnology, China Agricultural University, Beijing, China; ^2^College of Biological Sciences, China Agricultural University, Beijing, China; ^3^Beijing Research Center of Intelligent Equipment for Agriculture, Beijing Academy of Agriculture and Forestry Sciences, Beijing, China; ^4^Center for Crop Functional Genomics and Molecular Breeding, College of Agronomy and Biotechnology, China Agricultural University, Beijing, China

**Keywords:** gibberellin, maize, salt stress, water potential, osmotic adjustment, reactive oxygen species, vacuolar sodium sequestration

## Abstract

Salinity stress impairs plant growth and causes crops to yield losses worldwide. Reduction of *in vivo* gibberellin acid (GA) level is known to repress plant size but is beneficial to plant salt tolerance. However, the mechanisms of *in vivo* GA deficiency-enhanced salt tolerance in maize are still ambiguous. In this study, we generated two independent maize knockout mutant lines of *ent*-copalyl diphosphate synthase (one of the key enzymes for early steps of GA biosynthesis), *zmcps-1* and *zmcps-7*, to explore the role of GA in maize salt tolerance. The typical dwarf phenotype with lower GA content and delayed leaf senescence under salinity was observed in the mutant plants. The leaf water potential and cell turgor potential were significantly higher in *zmcps-1* and *zmcps-7* than in the wild type (WT) under salt stress. The mutant plants exhibited a lower superoxide anion production rate in leaves and also a downregulated relative expression level of NAPDH oxidase *ZmRbohA-C* than the WT maize under salt stress. Also, the mutant plants had higher enzymatic activities of superoxide dismutase (SOD) and catalase (CAT) and higher content of soluble sugars and proline under salt stress. The Na^+^/K^+^ ratio was not significantly different between the mutant maize plants and WT plants under salt stress conditions, but the Na^+^ and K^+^ content was increased in *zmcps-1* and *zmcps-7* leaves and shoots. Na^+^ fluorescent dye staining showed that the mutant leaves have significantly higher vacuolar Na^+^ intensity than the WT maize. The expression level of vacuolar Na^+^/H^+^ exchanger gene *ZmNHX1* and vacuolar proton pump genes *ZmVP1-1* and *ZmVP2* were upregulated in the *zmcps-1* and *zmcps-7* plants under salinity, further proving that *in vivo* GA deficiency enhanced vacuolar Na^+^ sequestration in *zmcps-1* and *zmcps-7* leaves cells to avoid Na^+^ cytotoxicity. Together, our results suggested that maintaining ROS homeostasis and enhancing vacuolar Na^+^ sequestration could be involved in GA deficiency-improved maize salt tolerance.

## Introduction

Soil salinity affects more than 800 million hectares of land, including 45 million hectares of irrigated land for agriculture (FAO, 2020). Due to the global climate changes and improper irrigation practices, the areas affected by salinity are expected to increase (Munns and Tester, 2008; Rengasamy, 2010). Not surprisingly, salinity has become a major abiotic stress limiting the sustainable production of crops and food security (Shabala, 2013). As glycophytes, most crops are sensitive to salt stress. Thus, improving crop salinity stress tolerance is of importance to meet the food supply demand from the increased population (Shabala, 2013).

Salinity inhibits plant growth mainly by osmotic stress, ion toxicity, and oxidative stress (Munns, 2002; Munns and Tester, 2008; Yang and Guo, 2018). To deal with the problem, plants have adapted in biochemical, physiological, and morphological properties to cope with salt stress (Munns and Tester, 2008). For example, plants can synthesize compatible osmolytes such as proline and soluble sugars to adjust cell water potential and keep membrane potential and stability under salt-induced osmotic stress (Apse and Blumwald, 2002; Szabados and Savoure, 2010; Guo et al., 2015). Overexpression of the proline biosynthesis-related gene *delta 1-Pyrroline-5-Carboxylate Synthetase* (*P5CS*) improves salt tolerance in grass plants (Guan et al., 2018). Meanwhile, high Na^+^ concentration in cytosol is harmful to plant growth, and maintaining a suitable cellular Na^+^/K^+^ homeostasis is critical to prevent cell damage and metabolic inhibition (Serrano et al., 1999). *SALT OVERLY* SENSITIVE1 (SOS1)-mediated Na^+^ exclusion and *NA^+^/H^+^ EXCHANGER1* (NHX1)-mediated vacuolar Na^+^ sequestration are considered as two important strategies for preventing Na^+^ cytotoxicity (Wu, 2018). Two high-affinity K^+^ channel (HKT) family genes *ZmHKT1* and *ZmHKT2* are identified to be involved in maize salt resistance by regulating root-to-shoot Na^+^ delivery and K^+^ accumulation in shoots (Zhang M. et al., 2018; Cao et al., 2019). Furthermore, the ionic toxicity and osmotic stress induce a secondary oxidative stress (Munns and Tester, 2008; Yang and Guo, 2018). And it is an effective way for plants to diminish oxidative stress to increase antioxidase activities and ROS scavenging (Zhu, 2002; Waszczak et al., 2018).

For survival under these stresses, plant hormones play essential roles in regulating biological function processes responding to various environmental conditions (Peleg and Blumwald, 2011). Although the abscisic acid (ABA) signaling pathway has been identified as a central regulator of abiotic stress response in plants, an increasing number of studies suggest that gibberellin acid (GA) signals are a priority in the plant growth response to abiotic stresses (Colebrook et al., 2014). For instance, drought stress affects GA metabolism to reduce GA content for repressing plant growth in maize, and emmer wheat drought-sensitive traits are associated with low *GIBBERELLIN 2-oxidase* (*GA2ox*) expression in roots (Wang et al., 2008; Krugman et al., 2011). Additionally, the roles of GA in plant growth regulation under abiotic stress are also supported by the discovery of the DELLA protein-mediated pathway in growth repression when suffering from various abiotic stresses (Achard et al., 2006; Achard et al., 2008a; Magome et al., 2008). Under salt stress, seed germination is delayed due to the repressed GA biosynthesis (Lopez-Molina et al., 2001). The overexpression of the *GA2ox* gene improves salt tolerance by reducing GA content in rice (Shan et al., 2014) and cotton (Shi et al., 2019). Meanwhile, overexpressed *DWARF AND DELAYED FLOWERING1* (*DDF1*) gene can upregulate the *GA2ox7* expression involved in increasing salt tolerance in *Arabidopsis* (Magome et al., 2008). Kim et al. (2008) observed that high salinity represses bioactive GA synthetic enzyme gene *GIBBERELLIN 3-oxidase1* (*GA3ox1*) expression, which is involved in an ABA-independent salt signaling pathway. Together, these results suggest that reducing GA content has a strong relationship with improving crop salt tolerance, but the physiological and biochemical mechanisms of *in vivo* GA reduction-improved salt tolerance are still unclear.

Maize is an important crop worldwide cultivated in aerobic soils and is recognized as a moderately salt-sensitive crop (Pitann et al., 2013). It is essential to understand the physiological and biochemical mechanisms in maize adapted to salt stress for breeding the salt tolerance varieties. In recent years, several studies have been reported on the mechanism of salt tolerance in maize with main focuses on ABA signal pathway. It has been identified that the ABA-dependent pathway to improve maize salt resistance is by upregulating or overexpressing calcineurin B-like protein gene *ZmCBL9* (Zhang F. et al., 2016), *PHYTOCHROME-INTERACTING FACTOR 3* (Gao et al., 2015), and bZIP transcription factor gene *ZmbZIP72* (Ying et al., 2012). Moreover, overexpression of *AtLOW OSMOTIC STRESS5* (*AtLOS5*) enhances ABA production and thus improves salt tolerance in maize (Zhang J. et al., 2016). Tuna et al. (2008) reported that exogenous application of GA_3_ can improve salt tolerance in maize via modulating the activities of antioxidases. On the contrary, genetic manipulation of GA metabolic genes improves salt tolerance in crops through reducing GA accumulation (Shan et al., 2014; Shi et al., 2019). Therefore, it is still largely unknown how GA signal plays a role in maize salt tolerance.

Gibberellin acid biosynthesis starts from trans-geranylgeranyl diphosphate through the sequential enzyme catalysis of two types of diterpene cyclases including *ent*-copalyl diphosphate synthase (CPS) and *ent*-kaurene synthase, followed by two membrane-associated P450 monooxygenases and then by families of GA 20-oxidase (GA20ox) and GA 3-oxidase (GA3ox). Finally, a family of GA 2-oxidase (GA2ox) is in charge of the action of GA inactivation (Yamaguchi, 2008; Hedden and Thomas, 2012). Here, the *zmcps* mutant was generated by clustered regularly interspaced short palindromic repeat-CRISPR-associated protein 9 (CRISPR-Cas9) knockout lines of *ZmCPS1* (*AN1*), which encodes the first diterpene cyclase in GA biosynthesis pathway (Zhang et al., 2020). The mutant showed a typical GA-deficient dwarf plant. Moreover, confocal imaging with fluorescent dye (CoroNa Green and APG-2) and atomic absorption spectrophotometer were used to analyze Na^+^ and K^+^ accumulation and distribution in maize leaf. The leaf water status, osmolyte content, superoxide anion production rate, and antioxidant enzyme activities were determined. Combined with the analysis of GA content, the physiological and biochemical mechanism of GA in modulating how maize responded to salt stress would be clarified.

## Materials and Methods

### Generation of the CRISPR-Cas9 Knockout Lines of the ZmCPS1

The CRISPR-Cas9 knockout lines of ZmCPS1 were created according to previous methods (Xing et al., 2014). Briefly, a pCAMBIA-derived CRISPR-Cas9 binary vector with two gRNA (as shown in Supplementary Figure S1) expression cassettes targeting two adjacent sites of *ZmCPS1* was generated and then transformed into *A. tumefaciens* strain EHA105. The following transformation process of infection of immature embryos from the B73-329 inbred line [wild type (WT)] was conducted by the Research Center for Functional Genomics and Crop Breeding of China Agricultural University. The genomic regions encompassing the gRNA-targeted sites were amplified and sequenced by Sanger sequencing to confirm the positive knockout lines. Two of these independent knockout mutant lines were used for this study: *zmcps-1*, conferring 1 bp insertion, and *zmcps-7*, conferring 4 bp deletion. Both mutations caused frameshifting and truncation of *ZmCPS1*. The targeted mutagenesis of *ZmCPS1* via CRISPR-Cas9 is shown in Supplementary Figure S1; primers used are listed in Supplementary Table S1.

### Plant Growth and Treatments

The T3 generation of CRISPR-Cas9 knockout mutant lines was used for this research. Plant seeds were surface-sterilized, geminated, and transplanted to the nutrient solution in 5 L plastic boxes fully in accordance with the previous protocols and equipment optimized by our laboratory (Zhang J. et al., 2016). Six plants each of both mutants and WT were grown in the same box in a growth chamber with a 14 h photoperiod, a temperature cycle of 25/30°C dark/light, 400 mmol m^–2^ s^–1^ irradiance from a high-voltage sodium lamp, and a relative humidity of 55–65%. The nutrient solution was changed every 3 days consequently until the plants were harvested. For exogenous application GA experiments, maize was treated with GA_3_ when transplanted until harvest. GA_3_ stock solution (0.1 mol L^–1^ GA_3_ in ethanol) was added into the nutrient solution to a final concentration of 10^–6^ mol L^–1^ when changing the nutrient solution every time. Half pots of each treatment were changed into the nutrient solution with NaCl when plants reached the three-leaf stage (when the third leaf was fully expanded). The NaCl treatment concentration of 125 mM was chosen for 50% inhibition rate in WT total biomass at 12 days after treatment according to the preliminary experiments. NaCl treatment continued for 12 days. Ten pots for each treatment were set as replicates.

### Sample Collections

Plant shoot and leaf samples were harvested at the same time at 0, 1, 3, 6, 9, and 12 days after treatment. Harvested plants were rinsed and gently washed with sterilized distilled water. To determine the dry matter accumulation, samples were 80°C oven-dried to a constant weight. The inhibition ratio of shoot dry weight was calculated by the ratio of dry weight under NaCl treatment to the dry weight under non-saline conditions on the same day. Six independent plants of each treatment were collected as biological replicates. Because the third leaves were regarded as the fully functional leaves and stayed alive during the assay period after NaCl treatment, these leaves were separated when collecting samples for fresh and dry weight kinetics, staining, physiological properties, and gene expression assays. The fresh samples for physiological properties and gene expression assays were frozen in liquid nitrogen rapidly and stored at −80°C until determination.

### GA Content Assay With LC-MS

Bioactive gibberellin GA_1_, GA_3_, and GA_7_ were measured as described by Liu et al. (2018) with modification. In brief, exactly measured 1.0 g of the fresh plant material powder was extracted twice under 4°C with 10 ml of MeCN shaking for 8 h followed by 13,000 r min^–1^ × 5 min centrifuge. The extract was dried with a nitrogen stream and reconstructed with 400 ml of MeOH solution (0.1% methanoic acid). The solution was filtered through a 0.22 mm filter membrane and subjected to HPLC–MS/MS analysis. Gibberellin analysis was performed on a quadrupole linear ion trap hybrid mass spectrometer (QTRAP 6500, AB SCIEX, Foster City, CA, United States) equipped with an electrospray ionization source coupled with an HPLC (Aglient 1290, Agilent Technologies, United States) using a poroshell 120 SB-C18 (Agilent Technologies, United States) column (2.1 × 150 mm; 2.7 mm). The injection volume was 2 ml. The inlet method was set as follows: The mobile phase A solvent consisted of methanol and 0.1% methanoic acid, and the mobile phase B solvent consisted of ultrapure water and 0.1% methanoic acid. Gradient: 0–1 min, 20% A; 1–9 min, 20% A–80% A; 9–10 min, 80% A; 10–10.1 min, 80% A–20% A; 10.1–15 min, 20% A. MS conditions were as follows: a spray voltage of 4500 V, and air curtain, nebulizer, and auxin gas pressures of 15, 65, and 70 psi, respectively. The atomizing temperature was 400°C. Gibberellins were detected in negative multiple reaction monitoring (MRM) mode. Each sample consisted of six replicates from independent experiments.

### Analysis of Leaf Water Status Parameters

The fresh third leaves of maize were separated equally along the midrib and used for measuring the leaf water potential and osmotic potential, respectively. Leaf water potential was determined following the process as described previously (Zhang J. et al., 2016) using a Scholander-type pressure chamber, SAPS II (Model 3115, Soil Moisture Equipment Corp., United States). The leaves’ osmotic pressure was determined using a vapor pressure osmometer (Vapor 5520, Wescor Inc., United States) following the tissue sap extraction method described by Callister et al. (2006). Three independent leaves were determined as biological replicates, and the values are the mean reads of two measurements for each sample according to the protocols.

### Leaf and Shoot Na^+^ and K^+^ Content Determination

The independent oven-dried samples were used for ion content determinations after dry weight determination. The ground dry material of shoots and leaves was extracted with 1 M HCl for 24 h shaking at 30°C; Na^+^ and K^+^ concentrations were determined with an atomic absorption spectrophotometer (SpectAA-50/55, Varian, Australia) according to previous studies (Mao et al., 1985; Peng et al., 2008). The total Na^+^ and K^+^ accumulation was calculated by the product of concentration and biomass.

### Cell Na^+^ and K^+^ Imaging and Content Measurement With Confocal Laser Scanning Microscope

Na^+^ and K^+^ content in the 3-day NaCl-treated leaf cells were determined using the green fluorescent Na^+^ dye CoroNa Green indicator (Invitrogen, United Kingdom) and fluorescent K^+^ dye Green-2 AM (APG-2, Abcam, United Kingdom) following the protocols described by Wu et al. (2018a, b). For leaf cell determination, 2 × 8 mm segments of the third leaf were cut, and the adaxial epidermis was peeled off carefully. The adaxial aspect of leaf segments should cling to the dyeing solution. After 2 h staining, fluorescent signals were captured using a confocal laser scanning microscope (Carl Zeiss LSM710, Germany) with 40× objective. To visualize the vacuoles definitely, FM4-64, staining both plasma and vacuolar membranes, was added up to 20 mM in the staining buffer and co-incubated with CoroNa Green or Green-2 AM for another 1 h before observation. The excitation wavelength was 488 nm, and detection was between 510 and 520 nm for CoroNa Green and APG-2, and 610 and 630 nm for FM4-64. A z-stacks model was conducted with 2 μm section thickness and 20 layers per sample. Finally, the layer with strongest fluorescence intensity was analyzed by the Java software ImageJ (v 1.49, NIH United States). The background values were deducted by measuring the empty region. The vacuolar Na^+^ and K^+^ concentration qualification followed the method described by Wu et al. (2015). An example is exhibited in Supplementary Figure S2. In brief, an interest line (IL) was drawn across the cells in the confocal image, and then the “Plot profile” function of ImageJ was used to get the fluorescence intensity of each pixel on the IL. The regions of vacuole and cytosol were identified according to the peaks of FM4-64. Then the average fluorescence intensity of CoroNa in the vacuole was calculated as the cell vacuolar Na^+^ concentration. The meaning of readings from 10 cells in five view images of each treatment was used for representing the vacuolar Na^+^ concentration.

### RNA Extraction and Real-Time qPCR Analysis

Total RNA was extracted from maize leaves and roots using the EASYspin rapid plant RNA extraction kit (Aidlab, China) following the instructions of the manufacturer. Reverse transcription was performed using a PrimeScript^TM^ RT reagent kit (TaKaRa, China), and then real-time quantitative RT-PCR was performed on a 7500 fast real-time PCR system (Applied Biosystems, Foster City, CA, United States) using TB Green^®^ Premix Ex Taq^TM^ II (Takara, China). A 2^–ΔΔ*Ct*^-based process was used to quantify relative gene expression level. The Actin gene was chosen as an internal control to normalize the data. Three independent samples were set as biological replicates, and three technical replicates were assayed for each sample. The primers used for real-time qPCR amplification are listed in Supplementary Table S1.

### Assay of Physiological and Biochemical Properties

Leaf electrolyte leakage representing membrane damage was determined by the protocols described by Verslues et al. (2006), and the final conductivity of the solution was recorded after autoclaving to represent the electrolyte content in leaves. Leaf chlorophyll content was determined by a chlorophyll meter (SPAD-502, Konic, Japan). The free proline concentrations were determined with sulfonic acid according to the method described by Chen et al. (2007). The soluble sugar content measurement was conducted with the anthrone method as described by Shi et al. (2015). Superoxide anion radical (O_2_•^–^) accumulation in leaves was stained by 0.1% NBT solution (50 mM PBS, pH = 7.0, with 0.1% Triton-X) for 8 h in the dark. The O_2_•^–^ production rate was determined with the protocol described by Chen et al. (2016). The activities of superoxide dismutase (SOD) and catalase (CAT) were assayed according to the protocols as described in maize studies (Jiang and Zhang, 2001; Tuna et al., 2008). These physiological parameters were calculated according to the soluble protein content. Three independent samples were set as biological replicates, and values of each sample were the mean of three-time assay under the same conditions.

### Statistical Analysis

Statistical analysis of all data was conducted with SAS software (V8, SAS Institute Inc., Cary, NC, United States). Multiple comparisons were corrected based on Fisher’s protected least significant difference (LSD), and Student’s unpaired *t*-tests were used for pairwise comparisons. *P*-values < 0.05 were considered significant.

## Results

### Salt Stress Altered GA Accumulation for Modulating Plant Growth

Salt stress significantly decreased the bioactive gibberellin molecules GA_1_, GA_3_, and GA_7_ in WT and two *ZmCPS1* mutant plants (Table 1). As expected, GA content in *zmcps-1* and *zmcps-2* plants was both lower than those in WT plants.

**TABLE 1:** Bioactive GA content in the leaves of wild-type and *ZmCPS1* knockout mutants (*zmcps-1* and *zmcps-7*) at 3 days after salt stress.

Treatment	Genotype	GA_1_ (μg g^–1^)		GA_3_ (μg g^–1^)		GA_7_ (μg g^–1^)	
0 mM NaCl	WT	0.075	a	0.072	a	0.186	a
	*zmcps-1*	0.037	c	0.049	c	0.165	b
	*zmcps-7*	0.035	cd	0.050	c	0.159	b
125 mM NaCl	WT	0.057	b	0.056	b	0.152	c
	*zmcps-1*	0.031	e	0.044	d	0.144	d
	*zmcps-7*	0.033	de	0.042	d	0.141	d

*Data are shown as mean of six replicates. Different letters in the same column represent significant difference protected with Fisher’s LSD at *P* < 0.05 (*n* = 6). GA, gibberellin acid; WT, wild type; LSD, least significant difference.*

**FIGURE 1:**
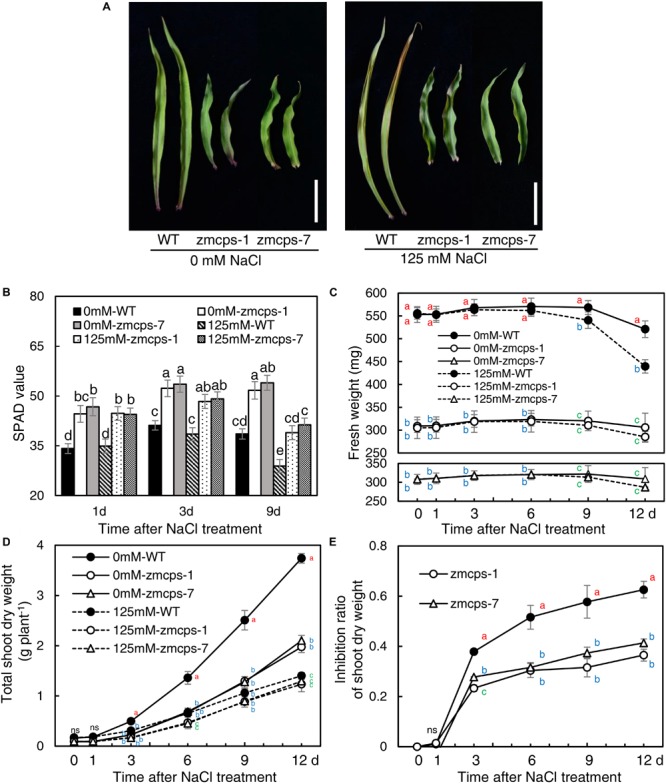
Leaf and shoot growth properties of wild-type (WT) and *ZmCPS1* knockout mutants (*zmcps-1* and *zmcps-7*) in response to salt stress. **(A)** The phenotype of the third leaves of *zmcps-1*, *zmcps-7*, and WT under salt stress (125 mM NaCl) and normal conditions (0 mM NaCl) for 9 days. White vertical bar = 5 cm. **(B)** Leaf SPAD values at 1, 3, and 6 days after salt treatment. Different letters represent significant difference protected with Fisher’s least significant difference (LSD) at *P* < 0.05. **(C)** The fresh weight kinetics of the third leaves under normal conditions and salt stress in 12 days. The values are means, and the vertical error bars are SD (*n* = 6). Different letters at the same time point represent significant difference protected with Fisher’s LSD at *P* < 0.05. ns, no significant difference. **(D)** Shoot dry weight kinetics and **(E)** inhibition ratio of shoot dry weight under salt stress of two maize lines. The values are means, and the vertical error bars are SD (*n* = 6). Different letters at the same time point represent significant difference protected with Fisher’s LSD at *P* < 0.05.

Because of the deficiency of GA, the two mutant plants, *zmcps-1* and *zmcps-7*, showed significant dwarf shoots with shorter and wider leaves, as well as smaller cell size, than the WT plant. The leaves of *ZmCPS1* mutant plants presented slighter wilting than the WT plant at 9 days after salt stress (Figure 1A and Supplementary Figures S3, S4). Although salt stress decreased the SPAD values in both the WT and the two mutant plants, the *zmcps-1* and *zmcps-7* plants had greater SPAD values than WT plants under both normal and salt stress conditions (Figure 1B). Meanwhile, the third leaf of the two mutant plants exhibited slight wilting, but that of the WT plant was moderately wilted. Correspondingly, the fresh weight of the third leaf presented a significant decrease in WT plants after 3 days under salt stress compared to normal conditions, while showing no significant difference in mutant plants between normal and salt stress conditions (Figure 1C). Besides, salt stress significantly repressed the dry weight accumulation of both WT and mutant plants (Figure 1D), and the effects of salt stress on plant biomass were greater in WT plants than the knockout mutant plants. In this case, the inhibition rate of salt stress to shoot biomass was 37.9–62.6% in WT plants, while it was 23.3–36.5 and 26.7–36.5% in *zmcps-1* and *zmcps-7* plants, respectively (Figure 1E).

Moreover, the WT and the mutants were treated with GA_3_ to test the effects of exogenous GA on maize salt tolerance (Supplementary Figures S5, S6). GA_3_ application significantly improved plant growth, and the leaves of both the WT and mutants were altered into a longer and thinner shape than control. The leaves’ wilting and tip senesce were more severe in the GA_3_-treated WT and mutants than the non-GA_3_-treated plants under salt tress. Although GA treatment increased the shoot dry weight of WT and mutant under both normal and salinity conditions, the inhibition ratio of shoot dry weight by salt ranged from 58.5% with control to 66.1% with GA_3_ treatment in the WT and from 45.4 to 58.9 and 44.6 to 58.6% in *zmcps-1* and *zmcps-7*, respectively.

### *In vivo* GA Deficiency Changed Leaf Water Status in Maize Seedlings Responding to Salt Stress

**FIGURE 2:**
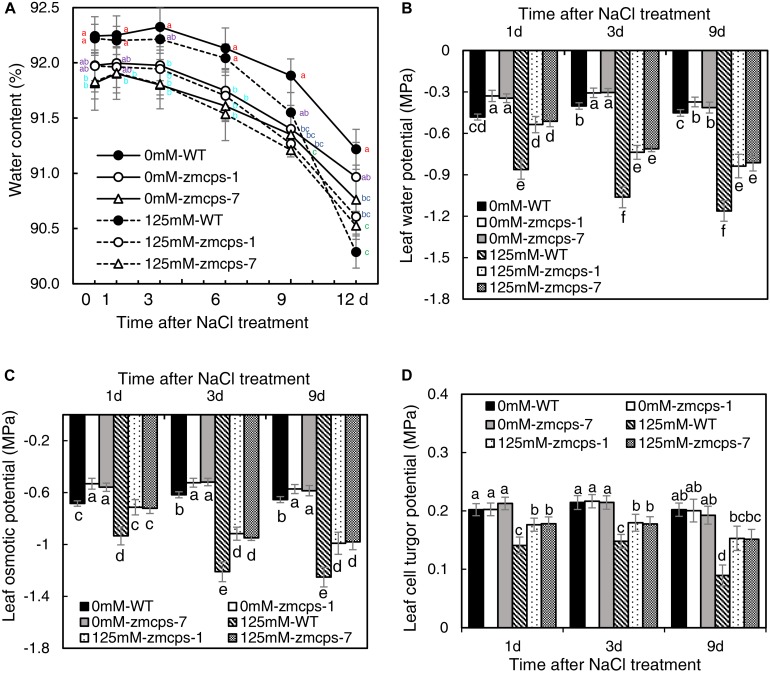
Leaf water status of *ZmCPS1* knockout mutants (*zmcps-1* and *zmcps-7*) and WT under salt stress. **(A)** The kinetics of leaf relative water content in 12 days under normal conditions (0 mM NaCl) and salt stress (125 mM NaCl). The values are means, and the vertical error bars are SD (*n* = 6). Different letters at the same time point represent significant difference protected with Fisher’s LSD at *P* < 0.05. **(B)** The leaf water potential, **(C)** the leaf osmotic potential, and **(D)** the leaf cell turgor potential of WT and mutant at 1, 3, and 9 days after salt stress. Values are means, and the vertical error bars are SD (*n* = 3). Different letters in each panel represent significant difference protected with Fisher’s LSD at *P* < 0.05.

Salt stress decreased the relative water content in WT and mutant leaves, and the *zmcps-1* and *zmcps-7* plants showed lower water loss for keeping higher relative water content compared to WT plants subjected to salt stress (Figure 2A). Moreover, the leaf water potential was significantly decreased by salt stress, and the *zmcps-1* and *zmcps-7* plants had higher leaf water potential than WT plants under both normal and salt stress conditions (Figure 2B). For example, the leaf water potential decreased by 0.59 MPa in WT leaves after 3 days under salt stress, while it decreased by 0.39 and 0.41 MPa in *zmcps-1* and *zmcps-7* leaves, respectively. Similar trends were observed in leaf osmotic potential; leaf osmotic potential in *zmcps-1* leaves was higher by 23.5, 24.2, and 20.9% than that in WT leaves at 1, 3, and 9 days after salt treatment, and results were similar in *zmcps-7* plants (Figure 2C). Meanwhile, salt stress significantly decreased the leaf cell turgor potential, and the two mutant leaves maintained higher cell turgor potential than the WT leaves at 3 and 9 days after salt stress (Figure 2D). Besides, there was no significant difference in cell turgor potential between the WT and two mutant leaves under normal conditions.

### *In vivo* GA Deficiency-Mediated Leaf ROS Balance and Plasma Membrane Stability Under Salt Stress

**FIGURE 3:**
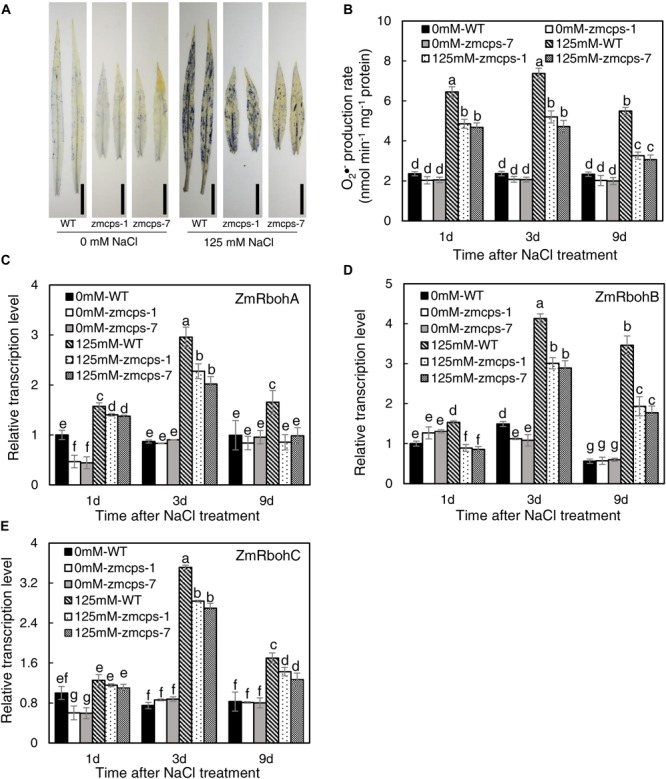
Plant superoxide anion radical (O_2_^–^) content and expression patterns of O_2_^–^ production enzyme genes *ZmRbohA* **(C)**, *ZmRbohB* **(D)**, and *ZmRbohC* **(E)** in the WT and *ZmCPS1* knockout mutants (*zmcps-1* and *zmcps-7*) under salt stress. **(A)** NBT staining of the third leaves of WT and mutants at 3 days after salt stress. Black vertical bar = 5 cm. **(B)** O_2_^–^ production rate of WT and mutants at 1, 3, and 9 days after salt stress. In each panel, values are means and the vertical error bars are SD (*n* = 3). Different letters in each panel represent significant difference protected with Fisher’s LSD at *P* < 0.05.

The NBT staining was conducted to detect the O_2_•^–^ content in WT, *zmcps-1*, and *zmcps-7* leaves, and salt stress boosted O_2_•^–^ accumulation in leaves (Figure 3A). Moreover, the O_2_•^–^ content in mutant leaves was lower than that in WT leaves. Consistently, salt stress significantly increased tissue O_2_•^–^ production rate, while the O_2_•^–^ production rate of *zmcps-1* and *zmcps-7* leaves was lower by 12.9, 17.7, and 30.3% and by 11.1, 14.9, and 29.1% than that of WT leaves at 1, 3, and 9 days after salt treatment (Figure 3B). Otherwise, the gene expression level of mainly O_2_•^–^ production enzyme, NAPDH oxidase ZmRbohA, ZmRbohB, and ZmRbohC, was also assayed, and salt stress significantly induced the expression of *ZmRbohA*, *ZmRbohB*, and *ZmRbohC* in WT and mutant leaves (Figures 3C–E). Meanwhile, the transcript expression of those genes in *zmcps-1* and *zmcps-7* leaves both presented lower than those in WT leaves under salt stress. For instance, the expression level of *ZmRbohA* was downregulated by 23.1 and 25.7% in *zmcps-1* and *zmcps-7* leaves compared to that in WT leaves at 3 days after salt stress treatment, and the values were 48.3 and 43.5% at 9 days after salt stress treatment. These three genes showed similar expression patterns. Moreover, the *zmcps-7* plants showed a generally lower trend than *zmcps-1*, while the difference between two mutant lines was not significant.

**FIGURE 4:**
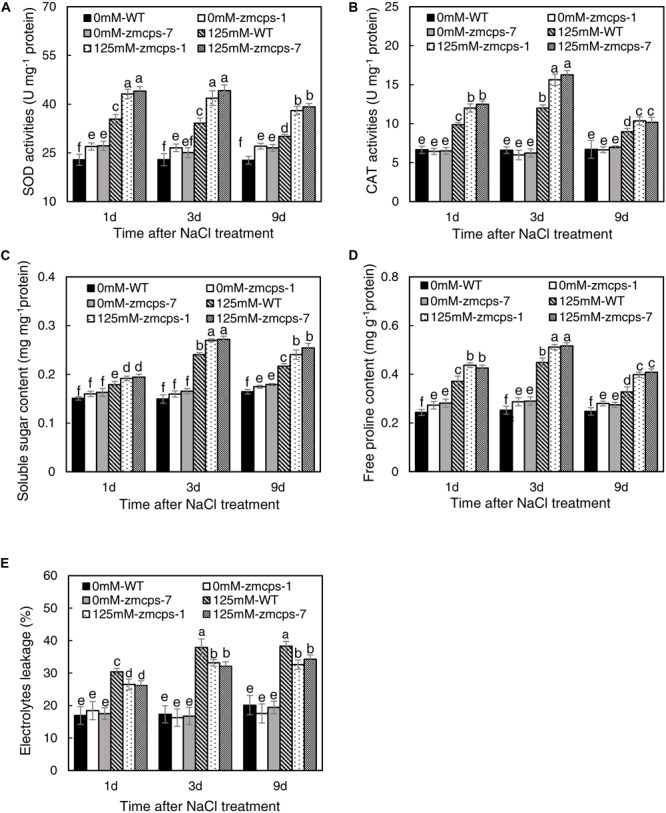
Activities of superoxide dismutase (SOD) **(A)** and catalase (CAT) **(B)**, soluble sugar content **(C)**, free proline content **(D)**, and electrolytes leakage **(E)** in leaves of WT and *ZmCPS1* knockout mutants (*zmcps-1* and *zmcps-7*) under salt stress. Values are means, and the vertical error bars are SD (*n* = 3). Different letters in each panel represent significant difference protected with Fisher’s LSD at *P* < 0.05.

The salt stress significantly enhanced the activities of SOD and CAT, and the *zmcps-1* leaves had higher SOD and CAT activities by 22.0–26.5 and 15.6–30.4% than WT leaves after salt stress treatment, respectively (Figures 4A,B). Furthermore, the activities of SOD were significantly increased by 15.9–19.0% in *zmcps-1* leaves compared to WT leaves under normal conditions. Similar trends were obtained in *zmcps-7* plants, and these two mutant lines showed no significant difference from each other. Generally, soluble sugar content was higher by 16.3–18.0 and 14.7–19.5% in *zmcps-1* and *zmcps-7* leaves than WT leaves under normal conditions (Figure 4C). Salt treatment significantly induced soluble sugar accumulation at 3 days after salt stress treatment, the soluble sugar content in *zmcps-1* leaves was increased, respectively, by 27.9 and 15.2% compared to WT leaves at 3 and 9 days after treatment, and *zmcps-7* plants exhibited similar results. Similar with soluble sugar content, free proline content was also improved significantly in *zmcps-1* and *zmcps-7* leaves under normal conditions (Figure 4D). Salt stress showed significant positive effects on leaf proline content, and the effects were obtained at 1 day after salt stress treatment. Moreover, the proline content was higher by 29.0–33.2 and 27.1–33.4% in *zmcps-1 and zmcps-7* leaves than WT leaves under salt stress, respectively.

As shown in Figure 4E, there was no significant difference in the relative electrolyte leakage between WT and *zmcps-1* and *zmcps-7* leaves under normal conditions. Moreover, the relative electrolyte leakage was obviously increased by salt stress, while it was higher by 14.2–17.4 and 12.1–16.8% in WT leaves than in *zmcps-1 and zmcps-7* leaves, respectively, after salt stress. Besides, the mutant leaves exhibited higher electric conductivity than WT leaves under normal and salt stress conditions (Supplementary Figure S8).

### *In vivo* GA Deficiency Adjusted Na^+^ and K^+^ Accumulation and Vacuolar Na^+^ Sequestration

**FIGURE 5:**
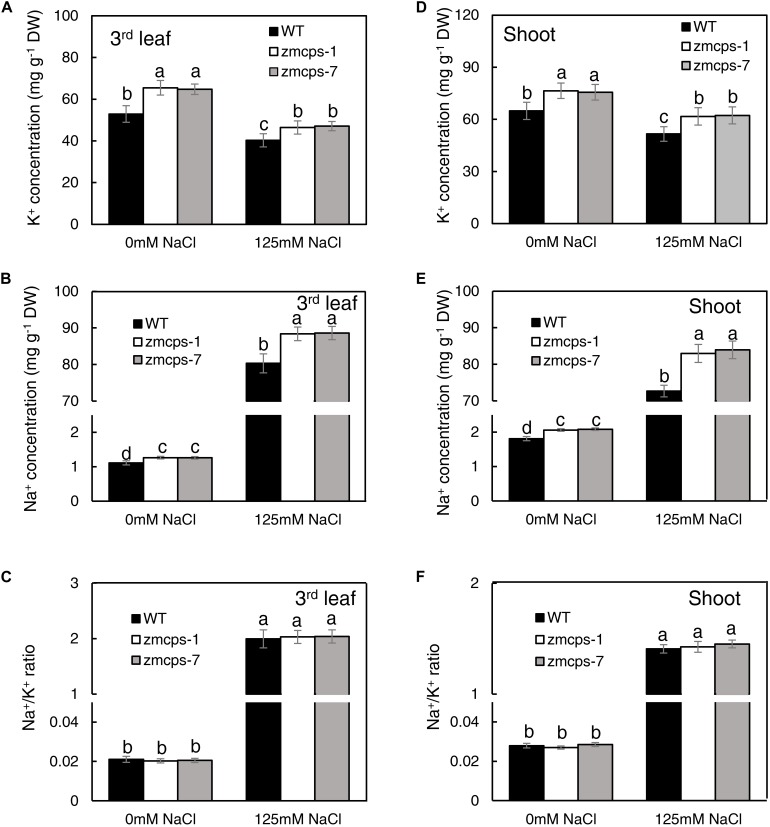
K^+^ **(A,D)**, Na^+^ **(B,E)** concentration, and Na^+^/K^+^ ratio **(C,F)** in leaves **(A–C)** and shoots **(D–F)** of WT and *ZmCPS1* knockout mutants (*zmcps-1* and *zmcps-7*) in response to salt stress. The values are means, and the vertical error bars are SD (*n* = 6). Different letters in each panel represent significant difference protected with Fisher’s LSD at *P* < 0.05.

Under normal conditions, the third leaf of *zmcps-1* and *zmcps-7* plants had higher K^+^ concentrations by 23.8 and 23.1% than that of the WT plant and had higher Na^+^ concentration by 13.5 and 13.4% than that of WT plant, and the Na^+^/K^+^ ratio in the third leaf of the two mutants presented no significant difference from the WT leaf (Figures 5A–C). When exposed to salt stress, Na^+^ concentration dramatically increased, while K^+^ concentration decreased in third leaf of both WT and mutant plants. Thus, the Na^+^ and K^+^ concentrations of the third leaf in *zmcps-1* plants were higher by 7.5 and 12.2% than those in WT plants, and results were similar in *zmcps-7* plants. Interestingly, there was no significant difference in Na^+^/K^+^ ratio between WT and mutant leaves under salt stress either. Meanwhile, similar changes were obtained in whole shoots under normal and salt stress conditions (Figures 5D–F). Salt stress enhanced the K^+^ and Na^+^ concentrations in WT and mutant shoots, and the *zmcps-1* and *zmcps-7* shoots both had greater K^+^ and Na^+^ concentrations than WT shoots under normal or salt stress conditions. Similarly, no significant difference was observed in Na^+^/K^+^ ratio between WT and mutant shoots regardless of normal or salt stress conditions.

**FIGURE 6:**
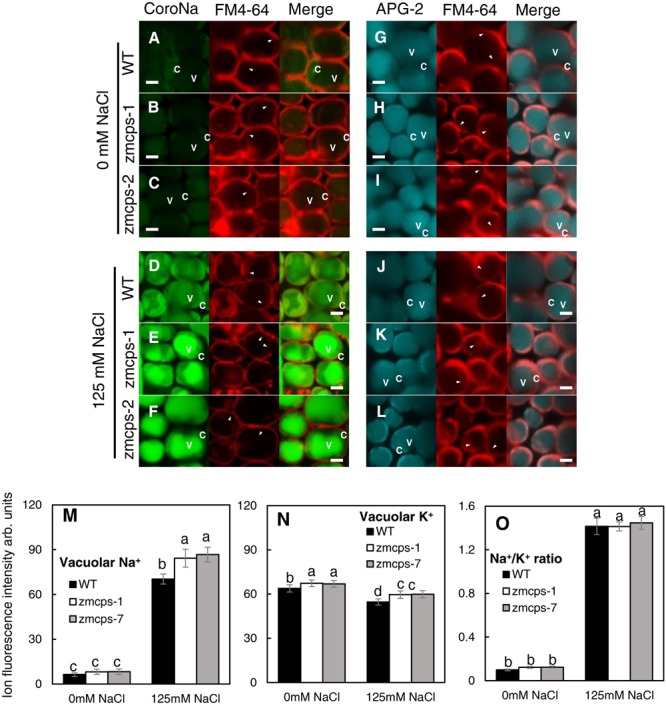
Comparison of Na^+^ and K^+^ fluorescence intensity in the WT and *ZmCPS1* knockout mutant (*zmcps-1* and *zmcps-7*) leaf cells at 3 days after salt stress (125 mM NaCl). **(A–F)** Image of leaf cells stained by CoroNa Green. **(G–L)** Image of leaf cells stained by Green-2 AM. **(A,D,G,J)** WT, **(B,E,H,K)** *zmcps-1*, **(C,F,I,L)** *zmcps-7*, **(A–C,G–I)** 0 mM NaCl, and **(D–F,J–L)** 125 mM NaCl treatment. White horizontal bar = 10 mm. **(M)** Statistic of fluorescence intensity of vacuolar Na^+^. **(N,O)** Statistic of fluorescence intensity of cell K^+^ and Na^+^/K^+^ ratio, respectively. The values are means, and the vertical error bars are SD (*n* = 50). Different letters in each panel represent significant difference protected with Fisher’s LSD at *P* < 0.05.

The mesophyll cell Na^+^ and K^+^ concentrations were detected with their specific fluorescent dyes, and similar results were obtained in *zmcps-1* and *zmcps-7* leaves. Salt stress significantly enhanced Na^+^ green fluorescent signal in the mesophyll cell vacuole of both WT and *zmcps-1 and zmcps-7* leaves (Figures 6D–F), while it decreased the K^+^ fluorescent signal in mesophyll cells of these three genotypes (Figures 6J–L). In addition, the fluorescence intensities of vacuolar Na^+^ and K^+^ were calculated, and vacuolar Na^+^ and K^+^ concentrations in leaf mesophyll cells were significantly higher in *zmcps-1* and *zmcps-7* plants than WT plants under normal and salt stress conditions (Figures 6M,N). Similarly, the Na^+^/K^+^ ratio in leaves was not significantly different between WT and mutant leaves (Figure 6O).

### *In vivo* GA Deficiency Modulated the Expression of Ion Transporter Related Genes Involved in Vacuolar Na^+^ Sequestration

**FIGURE 7:**
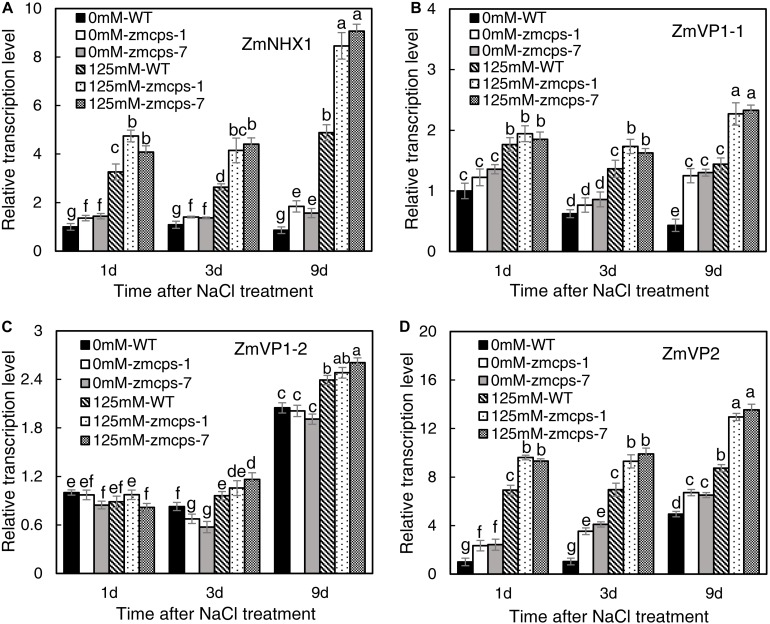
The expression patterns of vacuolar Na^+^ sequestration-related genes *ZmNHX1* **(A)**, *ZmVP1-1* **(B)**, *ZmVP1-2* **(C)**, and *ZmVP2* **(D)** in the WT and *ZmCPS1* knockout mutants (*zmcps-1* and *zmcps-7*) leaves under salt stress (125 mM NaCl). Values are means, and the vertical error bars are SD (*n* = 3). Different letters in each panel represent significant difference protected with Fisher’s LSD at *P* < 0.05.

Salt stress significantly increased the transcription level of Na^+^/H^+^ exchanger gene *ZmNHX1* (Figure 7A). The expression levels of *ZmNHX1* in mutant leaves were higher than those in WT leaves under normal and salt stress conditions, while the expression patterns of *ZmNHX1* were similar in *zmcps-1 and zmcps-7* leaves. In addition, the vacuolar proton pump V-type (vacuolar type) ATPase and the AVP1 H^+^-pyrophosphatase (PPase) genes *ZmVP1-1*, *ZmVP1-2*, and *ZmVP2* were also assayed. Salt stress significantly upregulated the expression of *ZmVP1-1* and *ZmVP2* in WT and mutant leaves, while *ZmVP1-2* was not affected significantly at the early stage under salt stress (Figures 7B–D). Meanwhile, the *zmcps-1* and *zmcps-7* plants maintained higher expression levels of *ZmVP1-1* and *ZmVP2* than WT plants under normal conditions and salt stress. The expression levels of *ZmVP1-2* were not significantly different in WT and mutant leaves under both normal and salt stress conditions.

## Discussion

Salt stress impairs crops production because of the reduction of plant growth (Munns and Tester, 2008). It is commonly observed that abiotic stresses induce lower a GA level for repressing the plant size, whereas genetic manipulation for reducing GA accumulation can improve stress resistance in several crops, including *Arabidopsis*, tobacco, rice, and cotton (Achard et al., 2006, 2008a,b; Colebrook et al., 2014; Shan et al., 2014; Shi et al., 2019). The CPS catalyzes the earlier step of GA biosynthesis in plants, and the loss-of-function mutant reduces the GA level, leading to a dwarf maize plant (Bensen et al., 1995; Yamaguchi, 2008; Hedden and Thomas, 2012). Similarly, in this study, the content of bioactive GA_1_, GA_3_, and GA_7_ decreased in maize under salt stress, and GA deficiency was observed in the dwarf mutants *zmcps-1* and *zmcps-7* (Table 1). Moreover, the maize CPS mutant plants showed delayed leaf senescence and maintained a lower inhibition ratio of shoot dry weight compared to the WT plants under salt stress (Figure 1), while plants of both WT and mutants with exogenous application of GA_3_ had severe leaf wilting under salinity and a higher inhibition ratio of shoot dry weight by salt compared to control (Supplementary Figures S6, S7). These results suggested that *in vivo* GA deficiency by mutation of *ZmCPS1* increased salt tolerance in maize.

Similar results were observed in rice (Shan et al., 2014) and cotton (Shi et al., 2019). Overexpression of the GA deactivation enzyme *GA2ox* genes improves the salt tolerance in these two species by reducing bioactive GA level. However, exogenous application of GA_3_ was also reported to increase maize salt tolerance by improving plant growth and nutrient uptake under salt stress (Tuna et al., 2008). Similar effects of GA_3_ application on increasing plant growth were observed in this study (Supplementary Figures S5, S6). But compared to control, the GA_3_-treated plants had a higher inhibition ratio of shoot dry weight by salt, which was not detected in the previous research. Besides, the cultivation method and NaCl and GA_3_ concentration applied were also different between the present study and the previous study. These differences of environment and application practices could lead to unstable plant phenotypes with GA and salt treatments. Thus, it could be better to use plants with constitutively regulated GA levels. And plant growth was inhibited by salt in many ways; to find which way is affected by GA will be beneficial to reveal the roles of GA in maize salt tolerance.

Osmotic stress is known as a component of salinity. Maintaining higher water potential is necessary for cell metabolism in plants to adapt to salt stress (Munns and Tester, 2008). In the present study, the *zmcps-1* and *zmcps-7* plants had higher leaf water potential and osmotic potential than WT plants under normal and salt stress conditions (Figure 2). This is beneficial for maintaining better water status and thus the lower inhibition of biomass accumulation in mutant plants under salt stress (Figure 1). Upon salinity stress, the primary adaptive strategy in plants is the regulation of osmotic potential. This can be done by modulating the production of osmolytes including soluble sugar and proline (Blumwald, 2003). For example, compared with sensitive ones, the salt-tolerant genotypes of grass crops including maize (Richter et al., 2015), rice (Boriboonkaset et al., 2013), and wheat (Kerepesi and Galiba, 2000) have high soluble sugar content. Also, overexpression of the proline biosynthesis enzyme gene *P5CS* increases the proline accumulation, which leads to improving salt tolerance in switchgrass (Guan et al., 2018). Here, the *zmcps-1* and *zmcps-7* plants both showed higher soluble sugar and proline content than the WT maize under salt stress. It is consistent with the result of higher osmotic potential in mutant leaves than the WT maize under salt stress. These results suggest that GA could modulate the biosynthesis of compatible osmolytes to maintain relatively higher leaf osmotic potential and better growth performance in maize under salt stress.

Salt ion toxicity is greatly attributed to the effects of Na^+^ and to the fact that that Na^+^ toxicity is related strongly to the allocation and distribution of K^+^ in plants (Shabala and Cuin, 2008; Anschütz et al., 2014). It is well accepted that the maintenance of intracellular K^+^ and Na^+^ homeostasis is critical for plant salt tolerance (Yang and Guo, 2018). In the current study, the *ZmCPS1* mutant plants exhibited higher salt tolerance, but their Na^+^/K^+^ ratio had no significant difference from WT plants under normal and salt stress conditions (Figure 5). Meanwhile, higher vacuolar Na^+^ and K^+^ concentrations were obtained in *zmcps-1* and *zmcps-7* plants compared to WT plants under salt stress conditions (Figure 6). These results suggested that GA deficiency enhanced the capacity of cell ion accumulation and tolerance to high cell Na^+^ concentration in maize. However, it is widely considered that high Na^+^ concentration damages cell metabolism under a saline environment. In some halophytes, regulating intercellular Na^+^ homeostasis is an important strategy to minimize cytotoxic effects of the ion and for osmotic adjustment (Munns and Tester, 2008). Increasing ion concentration in vacuoles could play roles in cell osmotic adjustment to counteract intracellular turgor reduction and maintain cell expansion (Hasegawa, 2013). Therefore, storing Na^+^ in the vacuole, which occupies the most of the mature cell volume, is an important mechanism in plant salt resistance. In this study, the fluorescent image using CoroNa Green Na^+^ dye showed higher vacuolar Na^+^ intensity in mesophyll cells of *zmcps-1* and *zmcps-7* leaves than the WT maize under salt stress conditions (Figure 6). These results indicate that GA deficiency could improve salt tolerance in maize through enhancing the ability of vacuolar Na^+^ sequestration.

The well-known Na^+^ transporter involved in vacuolar Na^+^ sequestration is the NHX1 Na^+^, K^+^/H^+^ exchanger (Wu, 2018). Overexpressing *OsNHX1* improved the rice cell survival rate through promoting Na^+^ into the vacuole under salt conditions (Fukuda et al., 2011). The importance of vacuolar Na^+^ sequestration in plants overcoming salt stress has been also proven in *Arabidopsis* (Apse et al., 1999) and tobacco (Gouiaa et al., 2012) by overexpressing the *NHX1* gene. In this study, the relative expression of *ZmNHX1* was significantly upregulated under salt stress (Figure 7A). This is similar to the previous study showing that the *ZmNHX* family members exhibit a dramatic salt-inducing expression pattern (Zorb et al., 2005). Meanwhile, the expression levels of *ZmNHX1* were significantly upregulated in both *zmcps-1* and *zmcps-7* plants compared to the WT plants under normal and salt conditions (Figure 6). Besides NHX1, vacuolar proton pump V-type ATPase and the vacuolar H^+^-PPase are also important for vacuolar Na^+^ sequestration, since they generate proton electrochemical gradients for energizing Na^+^ influx into vacuoles (Hasegawa, 2013). Here, we found that the mutant plants have significantly higher expression of the vacuolar proton pump genes *ZmVP1-1* and *ZmVP2* than the WT maize plants under salt stress (Figures 7B,D). These results suggest that GA deficiency modulated the transcript expression of *ZmNHX1* and *ZmVPs* to mediate Na^+^ influx into the vacuole in maize under salt stress, which in turn contributed to the higher salt tolerance of the mutant plants than the WT. How GA regulates cell vacuolar Na^+^ sequestration deserves further investigation.

ROS plays a crucial signaling role in plant response to salt stress (Zhu, 2002; Apel and Hirt, 2004), but the excessive accumulation of ROS causes damages on cell components such as the plasma membranes and chloroplast structure (Zhang Y.F. et al., 2018). O_2_•^–^ is one of the considerably long-lifetime ROS (ms level) in cells and is mainly produced by NADPH oxidase and scavenged by SOD (Waszczak et al., 2018). Here, the O_2_•^–^ level in *zmcps-1* and *zmcps-7* plants was significantly lower than WT plants under salt stress (Figure 3), suggesting the much healthier leaf of mutant plants under salinity. This is in agreement with the results of lower degrees of chlorophyll degradation (judged by leaf color, Figures 1A,B) and cell electrolyte leakage (Figure 4) in *zmcps-1* and *zmcps-7* plants under salt stress than the WT maize. Cell electrolyte leakage is an important indicator of plant salinity stress tolerance (Verslues et al., 2006). Consistent with the results of O_2_•^–^ production rate, compared with the WT maize, the mutant plants had lower expression levels of *ZmRbohs* and higher SOD and CAT activities (Figures 3, 4), indicating their good ability to scavenge O_2_•^–^ under salt stress. The osmolytes, proline and soluble sugars, are known to play important roles in ROS scavenging to resist multiple stress (Szabados and Savoure, 2010; Ben Rejeb et al., 2014). Besides improved O_2_•^–^ scavenging ability, the mutant plants also have significantly higher soluble sugars and proline (Figure 4), suggesting the synergies or coordination of antioxidant enzymes and osmotic adjustment in plants for salt stress tolerance. Similar with our findings, a previous study found that the gene expression levels of antioxidases, including SOD, POD, and CAT, were significantly increased in the *P5CS*-overexpressed plants compared to non-transgenic plants under salt stress (Guan et al., 2018).

## Conclusion

Salt stress significantly decreased bioactive GA content in both WT and *ZmCPS1* knockout mutant plants. The *zmcps-1 and zmcps-7* plants exhibited delayed leaf wilting and a lower inhibition rate of growth under salt stress compared to the WT. It was observed that Na^+^ and K^+^ concentration was increased in the leaf cell vacuoles of the two *ZmCPS1* mutants, but the Na^+^/K^+^ ratio was not significantly different between WT and mutant plants under both normal and saline conditions. The expression level of vacuolar Na^+^/H^+^ exchanger gene *ZmNHX1* and vacuolar proton pump genes *ZmVP1-1* and *ZmVP2* was upregulated in *zmcps-1* and *zmcps-7* leaves, which contributed to the high Na^+^ concentration in the vacuole of mutant leaf cells. Meanwhile, the osmolytes including soluble sugars and proline content were significantly increased in *zmcps-1* and *zmcps-7* plants, especially under salt stress. Consequently, the higher vacuolar Na^+^ concentration and accumulated osmolytes improved the osmotic potential in the mutant leaves to maintain higher water potential and turgor pressure under salt stress. Moreover, *zmcps-1* and *zmcps-7* plants had lower O_2_•^–^ accumulation than the WT maize under salt stress. This could be attributed to (1) the downregulated transcript level of NADPH oxidase genes *ZmRbohA-C*, (2) the increased enzymatic activities of SOD and CAT, and (3) the higher soluble sugars and proline in the mutant plants under salt stress. Overall, our results suggest that enhancing vacuolar Na^+^ sequestration and maintaining ROS homeostasis could be involved in GA deficiency-improved maize salt tolerance.

## Data Availability Statement

The datasets generated for this study are available on request to the corresponding author.

## Author Contributions

YZ, ZL, and MZ designed the experiments. YZ, JX, and JZ generated the maize knockout mutant lines. YZ, YW, JW, XLW, and XDW performed the experiments. YZ analyzed the data. YZ and MZ wrote the manuscript. All authors participated in the manuscript revision and approved the final manuscript.

## Conflict of Interest

The authors declare that the research was conducted in the absence of any commercial or financial relationships that could be construed as a potential conflict of interest.
