# Supplementary material for: Copalyl Diphosphate Synthase Mutation Improved Salt Tolerance in Maize (Zea mays. L) via Enhancing Vacuolar Na+ Sequestration and Maintaining ROS Homeostasis
Source: Front Plant Sci. 2020 May 13;11:457. doi: 10.3389/fpls.2020.00457 (PMC7237720; doi:10.3389/fpls.2020.00457)
Supplement: Supplementary file 1 [file Data_Sheet_1.PDF]

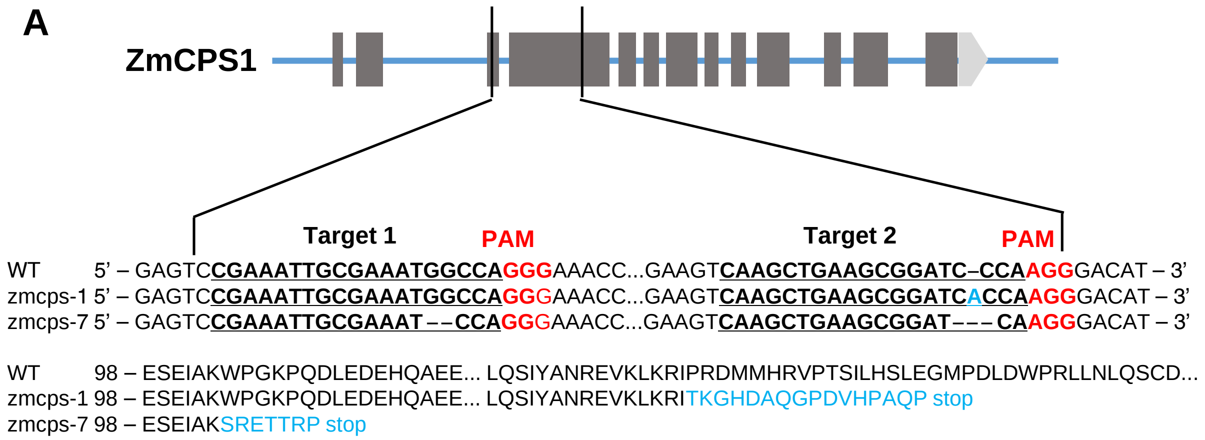

Supplementary Figure S1. Targeted mutagenesis of *ZmCPS1* via CRISPR/Cas9. Protospacer adjacent motif (PAM) sequences are labeled in red. gRNA target were underlined. The mutations in *zmcps-1* and *zmcps*-7 were shown.

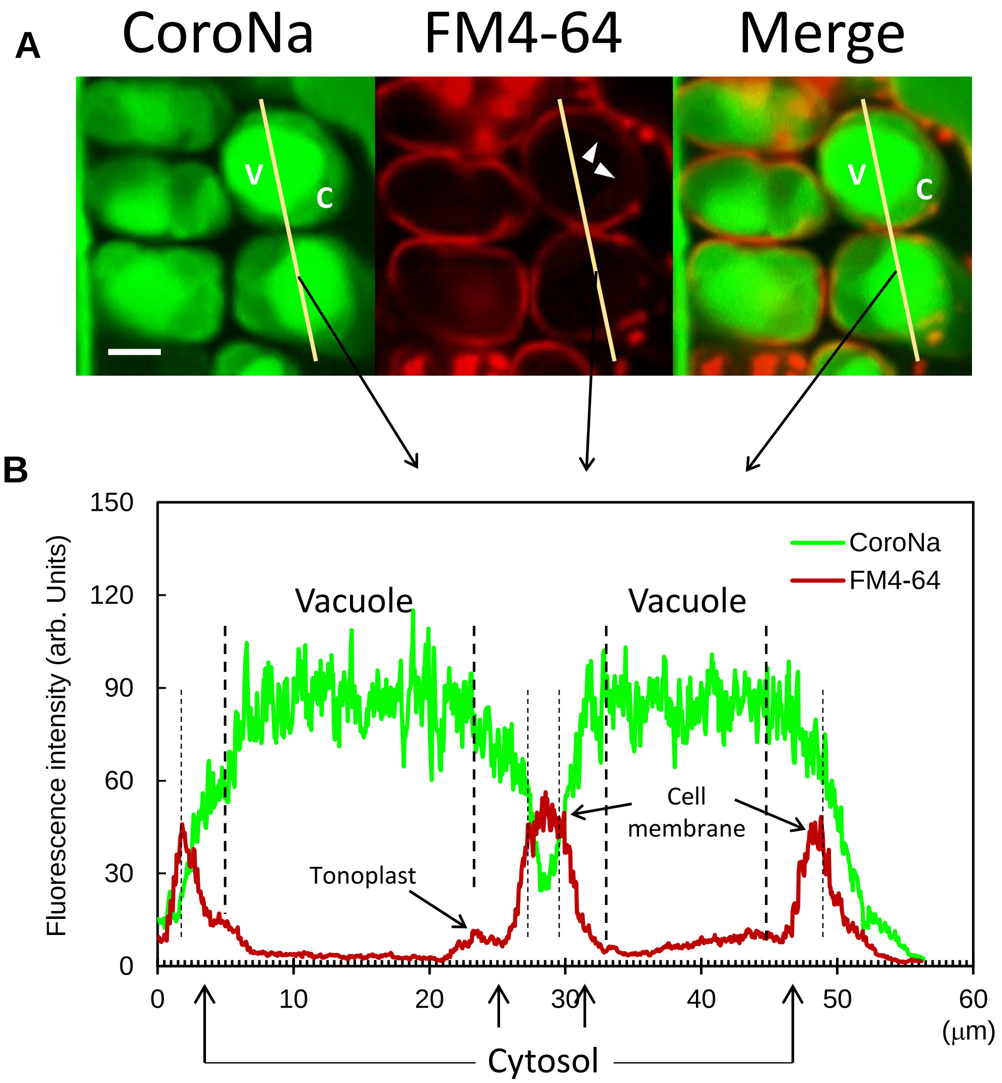

Supplementary Figure S2. Quantifying intracellular Na^+ distribution between the cytosol and the vacuole by double staining procedure. (A) Image of leaf cells stained by CoroNa Green and FM4-64. White bar = 10 µm. White horizontal bar = 10 mm. v for vacuole, c for cytosol. The white arrow pointed the tonoplast visualized by FM4-64. (B) The fluorescence intensity of CoroNa and FM4-64 along the interest line (IL, yellow solid line in panel A). The membrane was indicated by the peak of FM4-64 and then the vacuole region was identified. The vacuole Na^+ concentration was finally quantified.

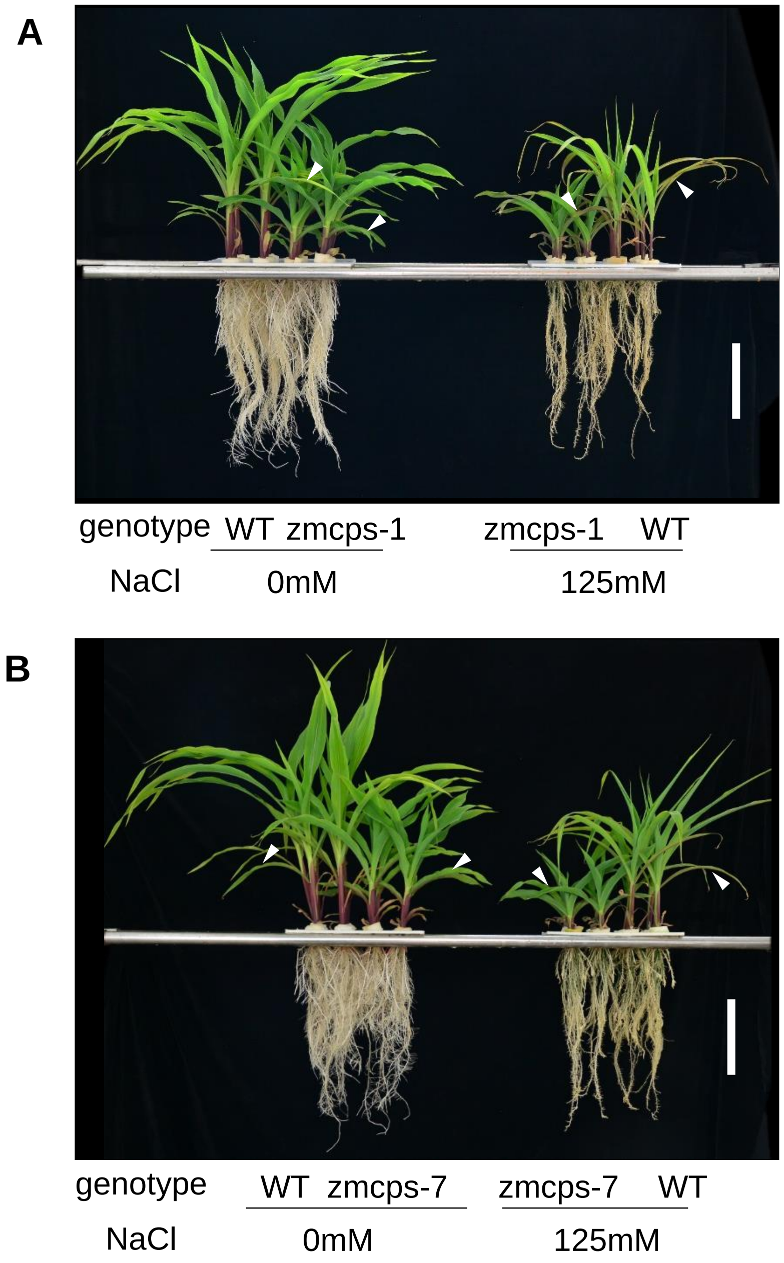

Supplementary Figure S3. Phenotype of wild-type (WT) and *ZmCPS1* knockout alleles (*zmcps-1* and *zmcps-7*) in response to salt stress at 9 d after salt stress. (A)WT and zmcps-1, (B) WT and zmcps-7. White arrowed pointed the third leaves. White vertical bar = 15 cm.

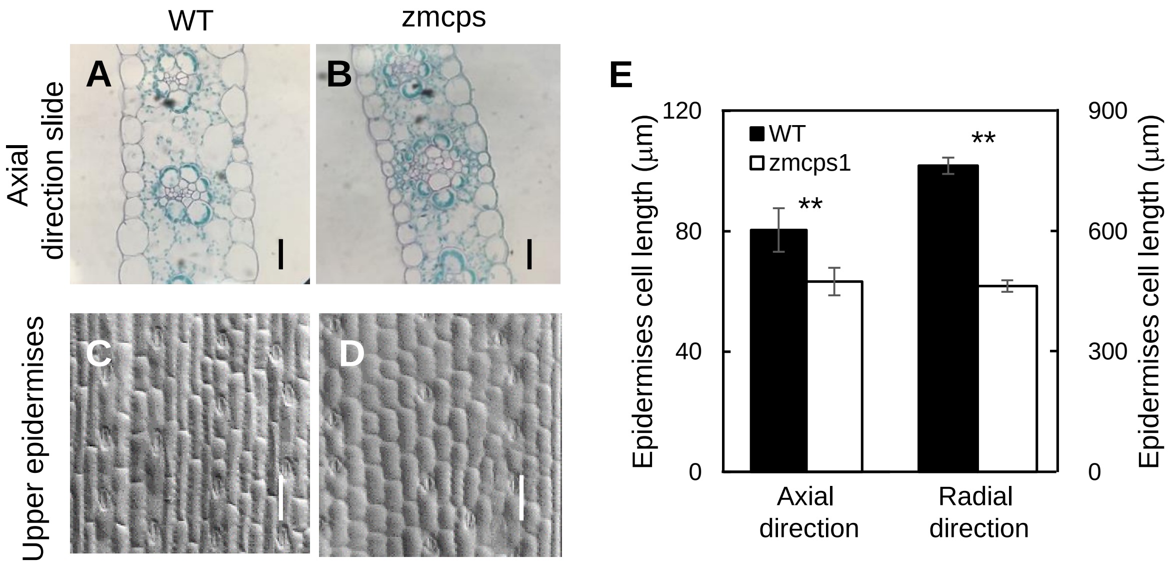

Supplementary Figure S4. Leaf cell size of wild-type (WT) and *ZmCPS1* knockout allele *zmcps-1*. (A, B) The paraffin section of the third leaf of WT and *zmcps-1*. Black vertical bar = 100 μm, (C, D) The scanning electron microscope image of upper epidermal the third leaf of WT and *zmcps-1* cells. White vertical bar = 500 μm. (C) Statistic of epidermal cell size, The values were means and the vertical error bar were SD (n=50). ** represented significant difference protected with Fisher's LSD at $P < 0.01$.

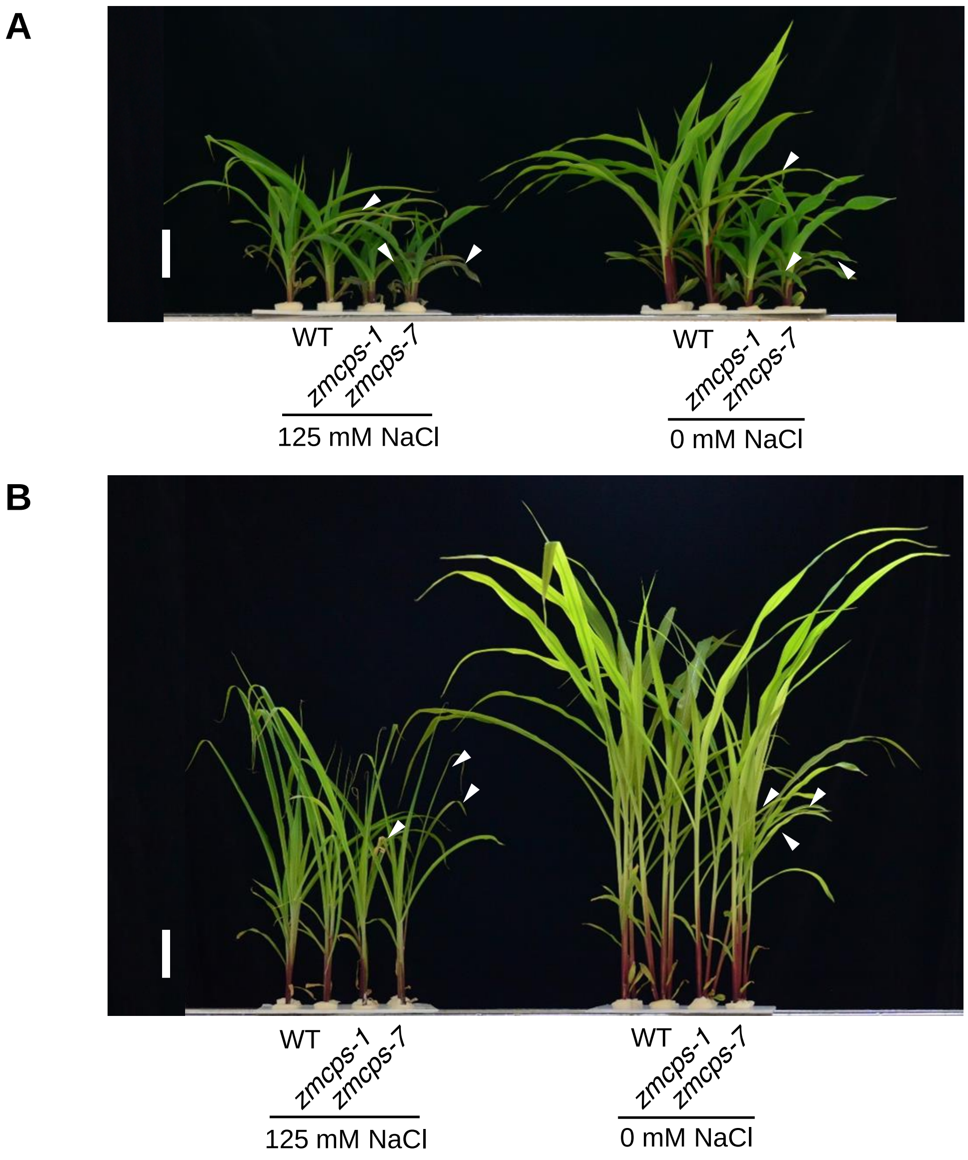

Supplementary Figure S5. Phenotype of wild-type (WT) and *ZmCPS1* knockout alleles (*zmcps-1* and *zmcps-7*) with GA_3 treatment in response to salt stress at 9 d after salt stress. (A)WT and knockout alleles under control, (B) WT and knockout alleles under GA_3 treatment. White arrowed pointed the third leaves. White vertical bar = 10 cm.

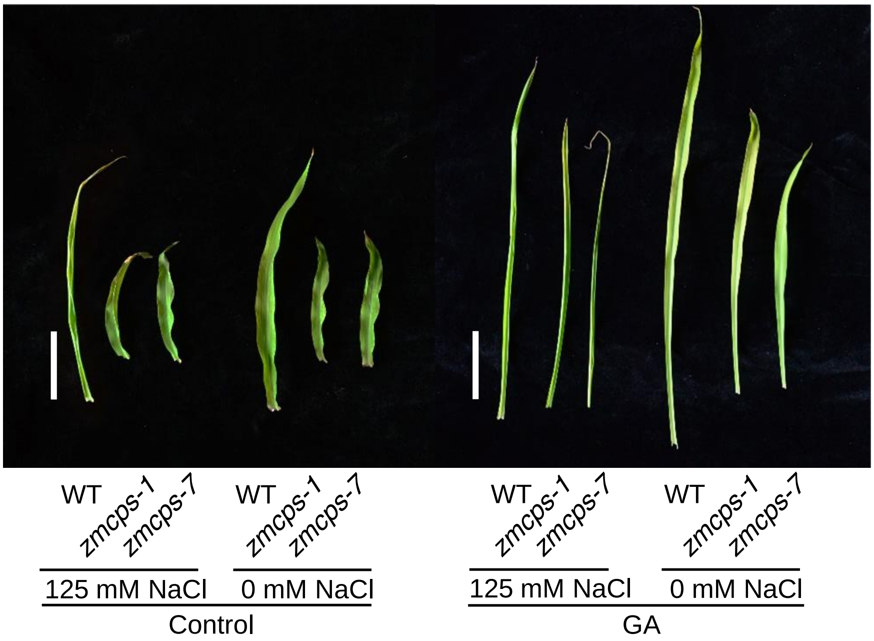

Supplementary Figure S6. The phenotype of the third leaves of wild-type (WT) and *ZmCPS* knockout alleles (*zmcps-1* and *zmcps-7*) with GA_3 treatment under salt stress (125mM NaCl) and normal condition (0mM NaCl) for 9d. White vertical bar = 5 cm.

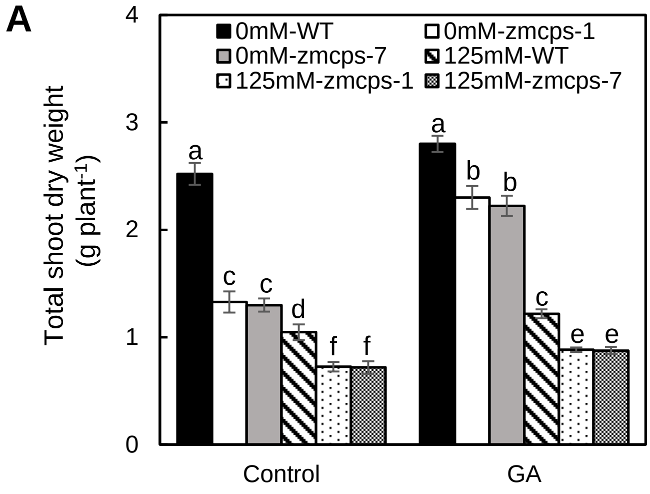

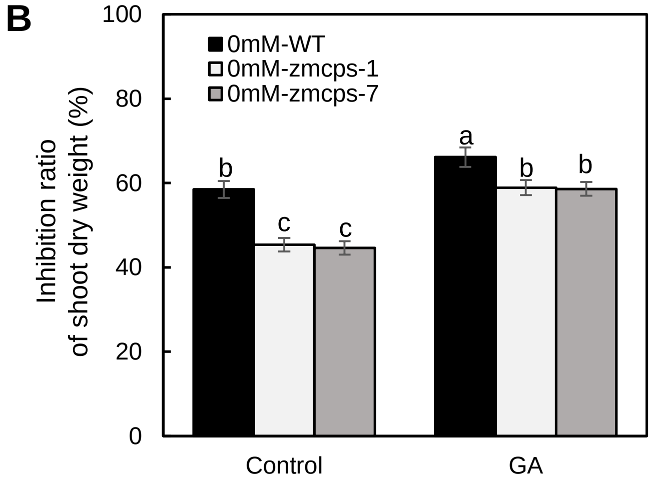

Supplementary Figure S7. Shoot dry weight (A) and inhibition ratio of shoot dry weight by salt (B) of wild-type (WT) and *ZmCPS* knockout alleles (*zmcps-1* and *zmcps-7*) with GA_3 treatment in response to salt stress for 9 days. The values were means and the vertical error bars were SD (n=6). Different letters in each panel represented significant difference protected with Fisher's LSD at $P < 0.05$.

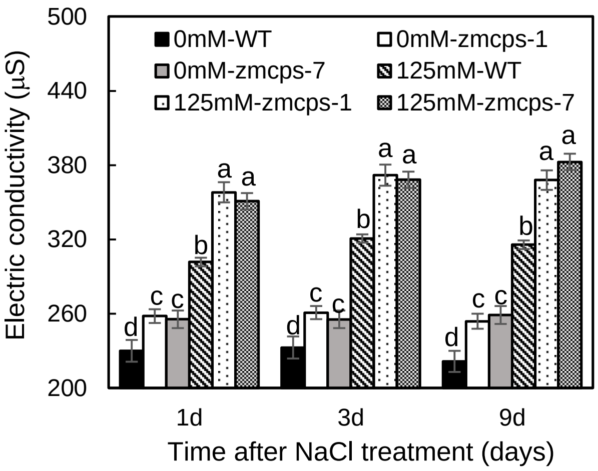

Supplementary Figure S8. Electric conductivity of the autoclaved leaf solution of in wild-type (WT) and *ZmCPS1* knockout alleles (*zmcps-1* and *zmcps-7*) leaves under salt stress. Values were means and the vertical error bar were SD (n=6). Different letters in each panels represented significant difference protected with Fisher's LSD at $P < 0.05$.

Supplementary Table S1, Primers used in this research.

**Experiment**			**Primer name**	**Forward primer sequence(5' to 3')**	**Reverse primer sequence(5' to 3')**
CRISPR-Cas9 gRNA-targeted site sequence			T1Seq	ATCAACCGTACTGCTGCAAACAG	CGCTAGCCTCCACATGTTCT
			T2Seq	AAGACCTTGAGGATGAGCACCAG	AAGTAGCGGGAGATCCCGAGT
**Experiment**	**Gene Name**	**Gene ID**	**Gene annotation**	**Forward primer sequence(5' to 3')**	**Reverse primer sequence(5' to 3')**
qRT-PCR	*ZmActin*	Zm00001d010159	actin1	GTTCCCTGGGATTGCCGAT	CTGCTGAAAAGTGCTGAG
	ZmRbohA	Zm00001d042961	respiratory burst oxidase A	ATGACATTCTTCTGCTTATTGGC	ATGCTTCCCACCTCTTCGTT
	ZmRbohB	Zm00001d043543	respiratory burst oxidase B	ACCCTTTGAATGGCATCCG	AAGGAGTTGCACCAATCCCTAAT
	ZmRbohC	Zm00001d038762	respiratory burst oxidase C	GAATACGAAAGCTGCACGGGCATT	CCAAAGTATTTGCGCAGTGGAGCA
	ZmNHX1	Zm00001d022504	Na+/H+ antiporter	GTTAACGAGTCCATCACCGC	CCGGCATTGAAGATGATGGG
	ZmVP1-1	Zm00001d015569	vacuolar proton pump homolog 1 (vpp1)	CTGGGACAACGCCAAGAAGT	AGCACATGCCATAGATGCTG
	ZmVP1-2	Zm00001d037492	inorganic H+ pyrophosphatase	AACCCTCTTCGGTGTTGAGA	GATTTGGCAGGAGTTATTCG
	ZmVP2	Zm00001d030996	H+-translocating pyrophosphatase	GGAATGTGGGTGTCTGTGCG	ACGAGGATCATCTTCGGGTA
